# Draft Genome Sequence of Pediococcus pentosaceus MZF16, a Bacteriocinogenic Probiotic Strain Isolated from Dried Ossban in Tunisia

**DOI:** 10.1128/MRA.00285-19

**Published:** 2019-04-25

**Authors:** Mohamed Zommiti, Amine M. Boukerb, Marc G. J. Feuilloley, Mounir Ferchichi, Nathalie Connil

**Affiliations:** a Unité de Protéomique Fonctionnelle et Potentiel Nutraceutique de la Biodiversité de Tunisie, Institut Supérieur des Sciences Biologiques Appliquées de Tunis, Université de Tunis El-Manar, Tunis, Tunisia; b Laboratoire de Microbiologie Signaux et Microenvironnement (LMSM) EA 4312, Université de Rouen Normandie, Normandie Université, Évreux, France; Indiana University, Bloomington

## Abstract

Pediococcus pentosaceus strain MZF16 was isolated from dried ossban, a Tunisian dry fermented meat. The MZF16 chromosome consisted of 28 contigs with a total draft genome size of 1,928,373 bp and a G+C content of 37.2%. This is the first genome characterization of a P. pentosaceus strain isolated from traditional Tunisian meat.

## Abstract (precis)

Pediococcus pentosaceus strain MZF16 was isolated from dried ossban, a Tunisian dry fermented meat. The MZF16 chromosome consisted of 28 contigs with a total draft genome size of 1,928,373 bp and a G+C content of 37.2%.

## ANNOUNCEMENT

Pediococcus pentosaceus is a Gram-positive, coccus-shaped, nonmotile, and non-spore-forming lactic acid bacterium (LAB) that is frequently isolated from dairy and meat products, bacterially ripened cheese, and plant materials. These strains are used in industry as starter cultures for natural and controlled fermented foods (1–4). They have also been described as reliable candidates for food preservation, as they produce bacteriocins (5, 6) and possess interesting probiotic potential (7). Here, we report the draft genome sequence of P. pentosaceus strain MZF16, recently isolated from dried ossban, an artisanal Tunisian dried meat prepared with sheep intestine, lung, heart, and meat cut into pieces and well mixed with salt and local spices. This original biotope was reported for the first time in two investigations led by Zommiti et al. (6, 8). P. pentosaceus MZF16 is a promising strain for use in biopreservation, as it has been shown to produce a bacteriocin, pediocin MZF16, that is highly active against Listeria monocytogenes and is 100% identical to coagulin (6).

Genomic DNA of P. pentosaceus MZF16 was extracted from overnight culture in MRS broth with the GeneJet genomic DNA purification kit (Thermo Scientific, France). Input DNA (1 ng as quantified by a double-stranded DNA [dsDNA] high-sensitivity kit on a Qubit fluorometer [Thermo Fisher Scientific, USA]) was used to prepare genomic libraries for sequencing using a Nextera XT DNA sample kit per the manufacturer’s protocol (Illumina, San Diego, CA). Libraries were sequenced on the Illumina MiSeq platform (LMSM Evreux, Rouen Normandy University, France) using a paired-end protocol (2 × 250 bp).

Paired-end reads were trimmed using Trimmomatic v.0.36 with a sliding-window quality cutoff of Q20 (9). Version 0.11.6 of FastQC (10) was used to check the quality of the 1,693,634 generated reads. Assembly of paired-end reads was done *de novo* using Unicycler v.0.4.7 with default settings (11). Quast v.5.0.0 was used to check assembly consistency, such as the number of contigs, G+C content, *N*_50_ value, and the total size (12). Annotations were done using the Prokka pipeline v.1.13.4 with default parameters (13). The multilocus sequence typing (MLST) profile of MZF16 isolate was determined from the draft genome sequence using the software package MLST v.2.16.1 (https://github.com/tseemann/mlst) (14) based on the Pediococcus pentosaceus PubMLST database (https://pubmlst.org/ppentosaceus/).

The assembled genome size was 1,928,373 bp with a G+C content of 37.2%, which was consistent with those of published P. pentosaceus genomes (https://www.ncbi.nlm.nih.gov/genome/genomes/1109). The Unicycler assembler yielded 28 contigs with an *N*_50_ value of 364,301 bp and a largest contig size of 435,252 bp (using only contigs larger than 200 bp). The draft genome had 1,924 predicted coding sequences, 54 tRNA genes, and 3 rRNA operons. The 16S rRNA gene showed 100% identity with that of P. pentosaceus strain SL4 (15) according to a BLAST search with the “align two or more sequences” option (https://blast.ncbi.nlm.nih.gov/). MLST analysis identified known allele numbers for *pyc-4*, *pgm-2*, and *pgI-6,* while previously undescribed alleles were detected for *gyrB*, *leuS*, *glnA*, and *dalR* genes (sequences were submitted to the Pediococcus pentosaceus PubMLST database on 5 March 2019).

### Data availability.

The draft genome sequence of P. pentosaceus MZF16 obtained in this whole-genome shotgun project has been deposited at DDBJ/ENA/GenBank under the accession number SKCK00000000. The version described in this paper is the first version, SKCK01000000. The raw sequencing data have been deposited in the same database under the accession number SRR8717133.
